# Supplementary material for: Longitudinal Immunoprofiling of the CD8+ T-Cell Response in SARS-CoV-2 mRNA Vaccinees and COVID-19 Patients
Source: Vaccines (Basel). 2025 May 22;13(6):551. doi: 10.3390/vaccines13060551 (PMC12197383; doi:10.3390/vaccines13060551)
Supplement: Supplementary file 1 [file vaccines-13-00551-s001.zip › vaccines-3608762-supplementary.pdf]

SUPPLEMENTARY MATERIAL

Supplementary Figures:

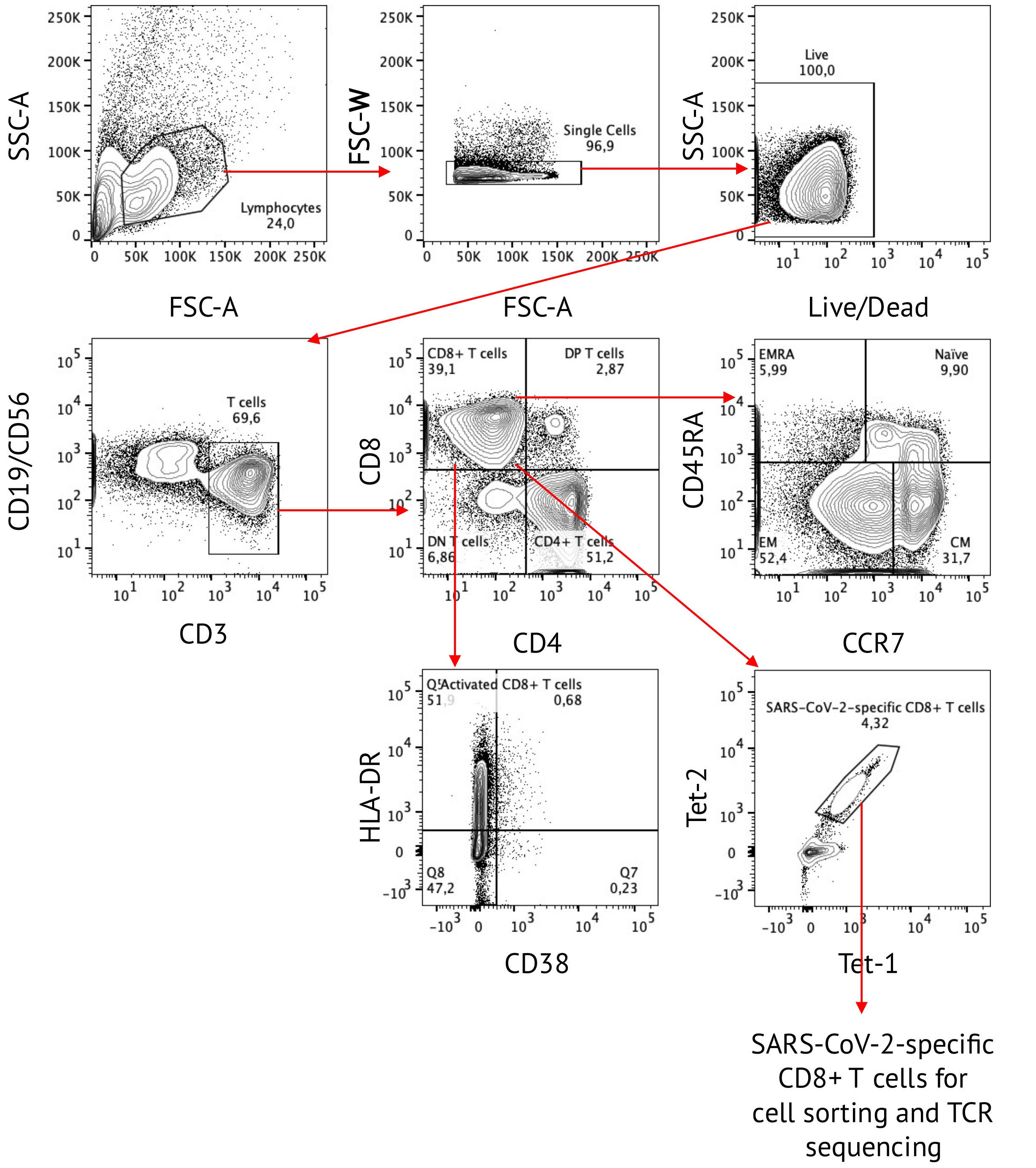

Suppl. Fig. S1. Gating strategy for activation and memory CD8$^+$ T cell profile and for SARS-CoV-2-specific CD8$^+$ T cells. Shown are representative statistics from a single COVID-19 patient. SSC stands for side scatter; FSC, forward scatter; FSC-H, forward scatter height; and FSC-W, forward scatter width.

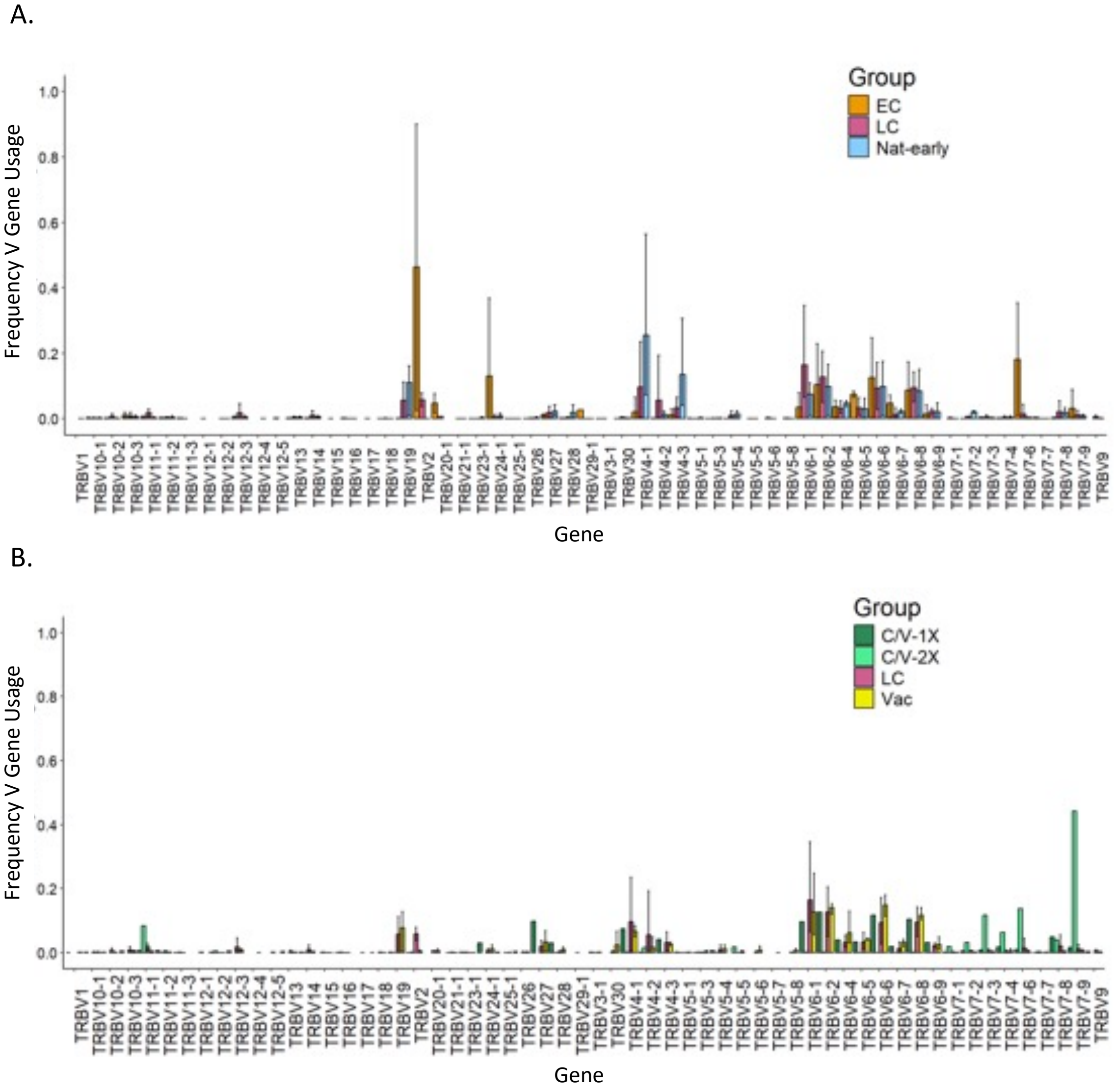

Suppl. Fig. S2. Usage of the V gene in different experimental groups. V gene usage for the CDR3 region was assessed for each group of patients through TCR sequencing. A: Comparison of V gene usage between Nat-early, EC and LC groups. B: Comparison of V gene usage between LC, C/V-1X, C/V-2X and Vac groups.

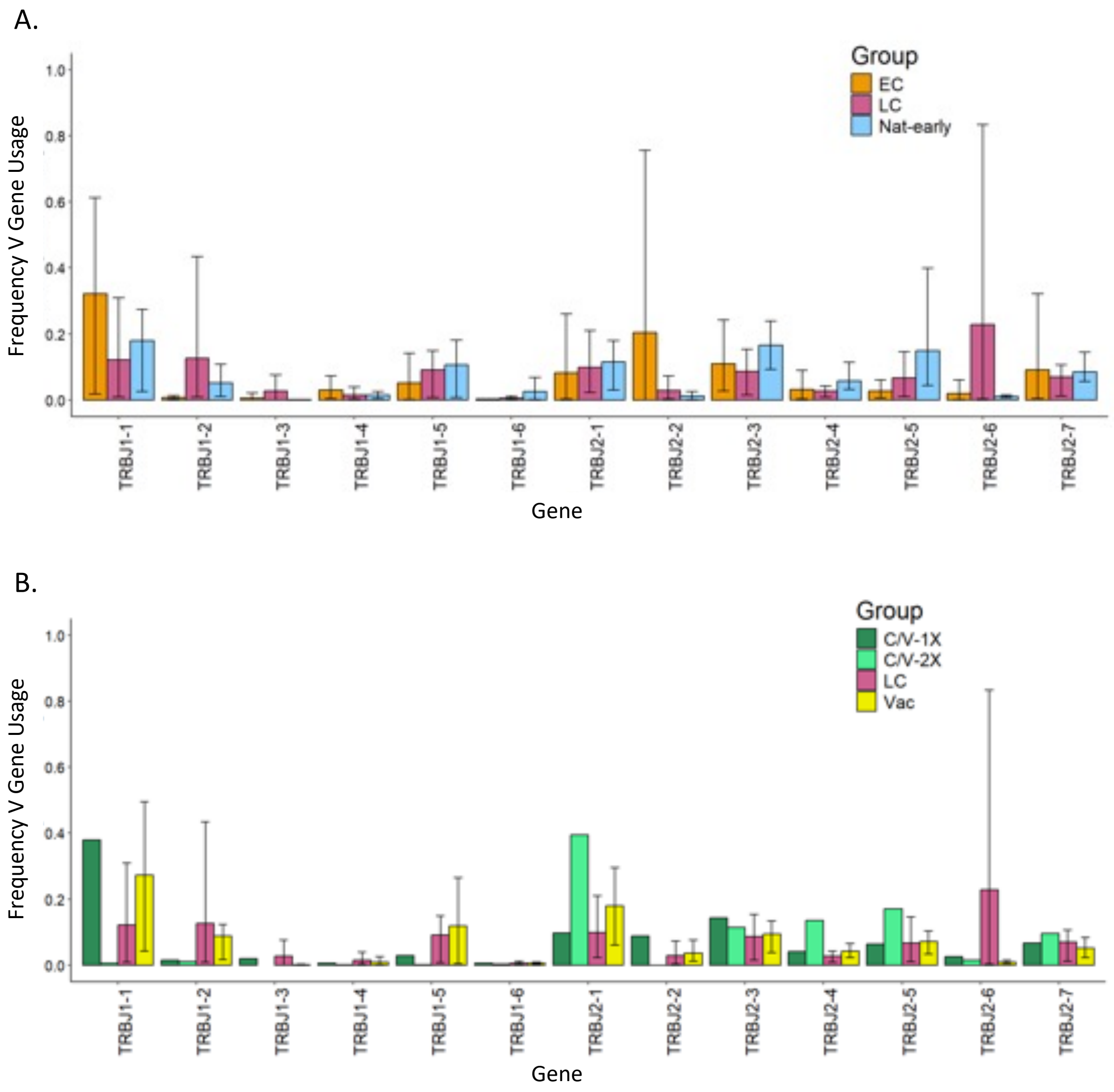

Suppl. Fig. S3. Usage of the J gene in the different experimental groups. J gene usage for the CDR3 region was assessed for each group of patients through TCR sequencing. A: Comparison of J gene usage between Nat-early, EC and LC groups. B: Comparison of J gene usage between LC, C/V-1X, C/V-2X and Vac groups.

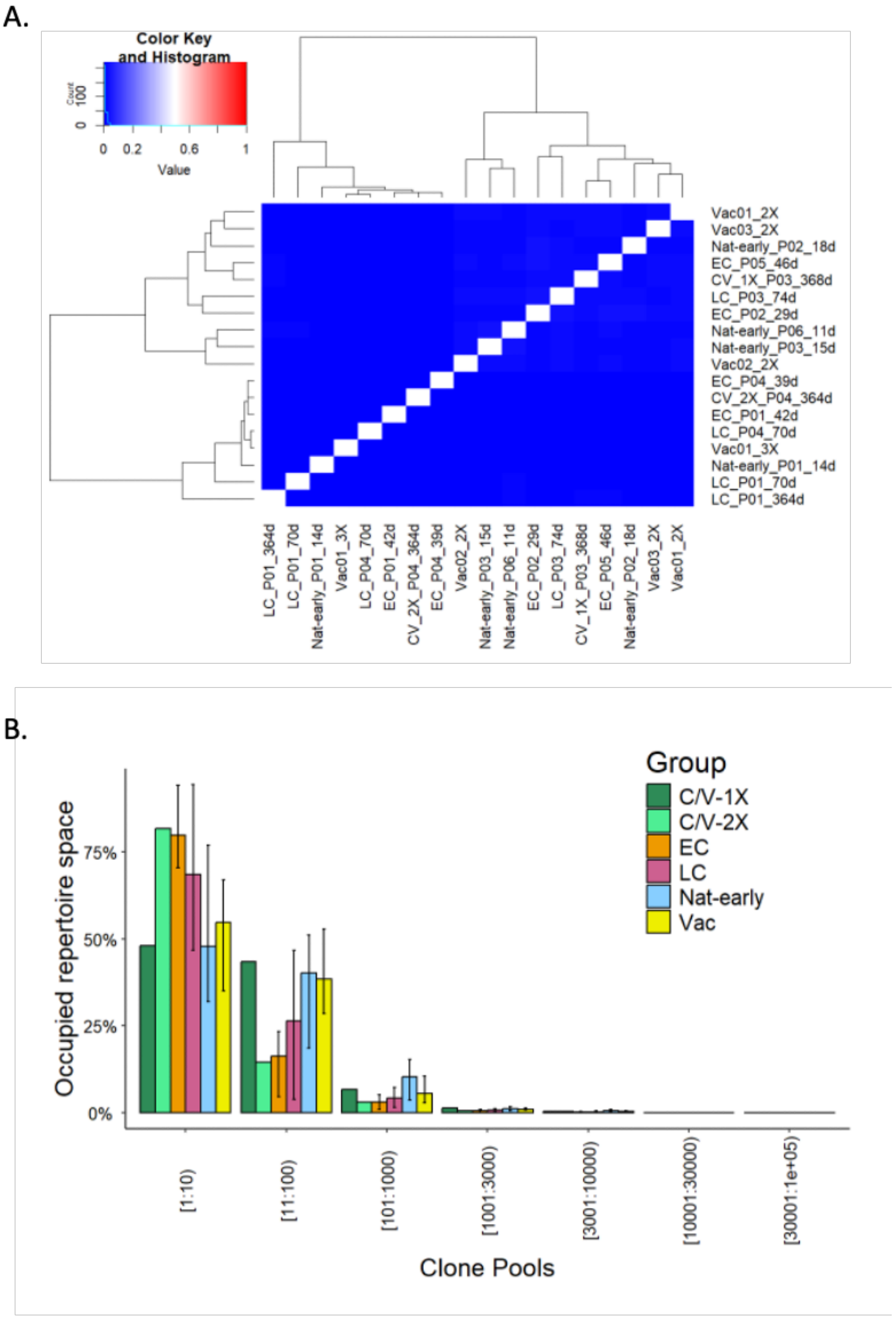

Suppl. Fig. S4. TCRβ CDR3s overlap and clonality proportion across COVID-19 patients and mRNA vaccines. (A) Heat map of overlapping indices across patients, measured through the Jaccard index. (B) Occupancy of TCR repertoire in patients by different number of clonotypes.

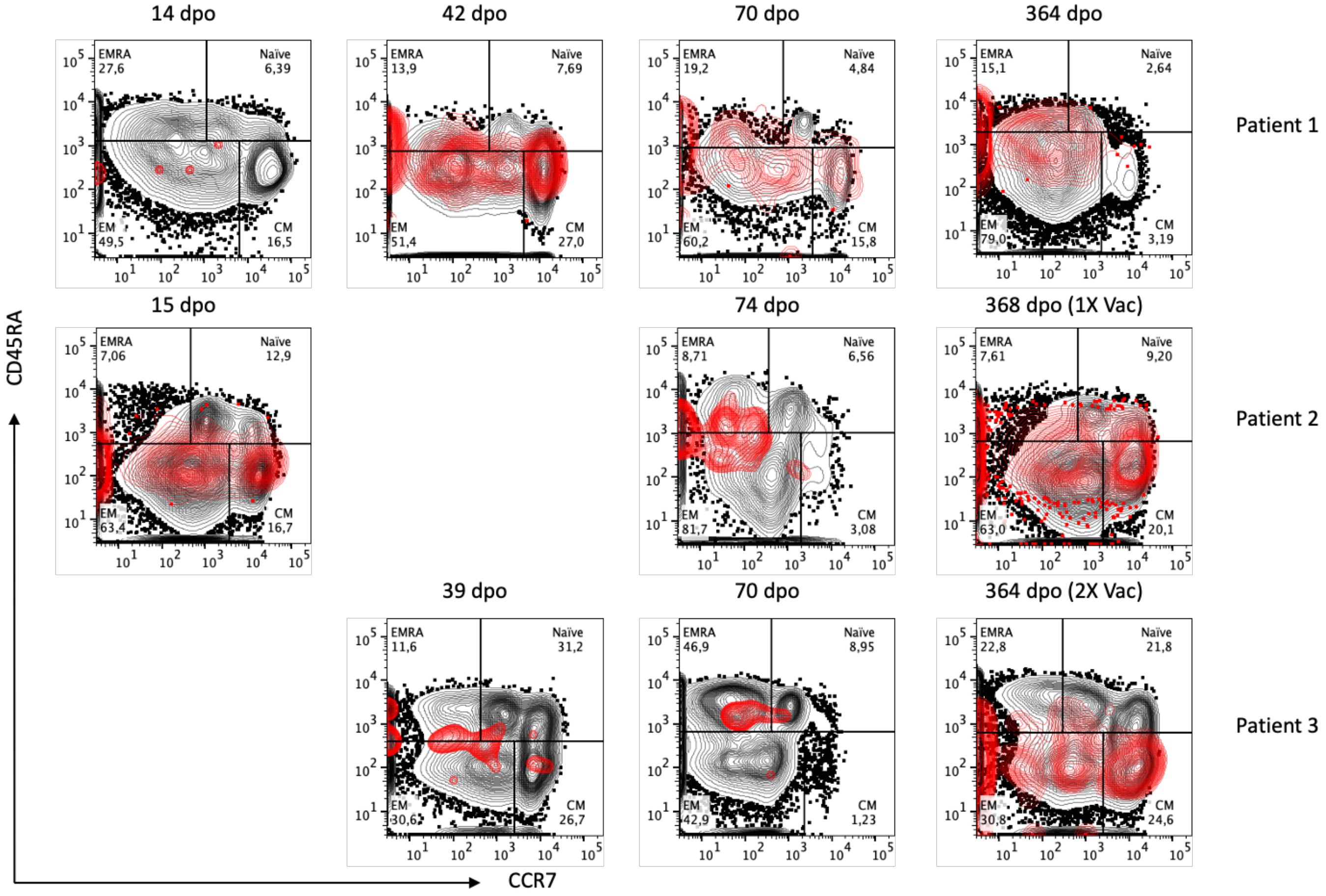

Suppl. Fig. S5. Evolution over time of the CD8$^+$ T cell memory profile. Evolution along the time of the CD8$^+$ T-cell memory profile (black) and SARS-CoV-2-specific CD8$^+$ T cells (red) within each subset. Day PI = days post-infection. 1x = one vaccination dose, 2x = two vaccination doses.

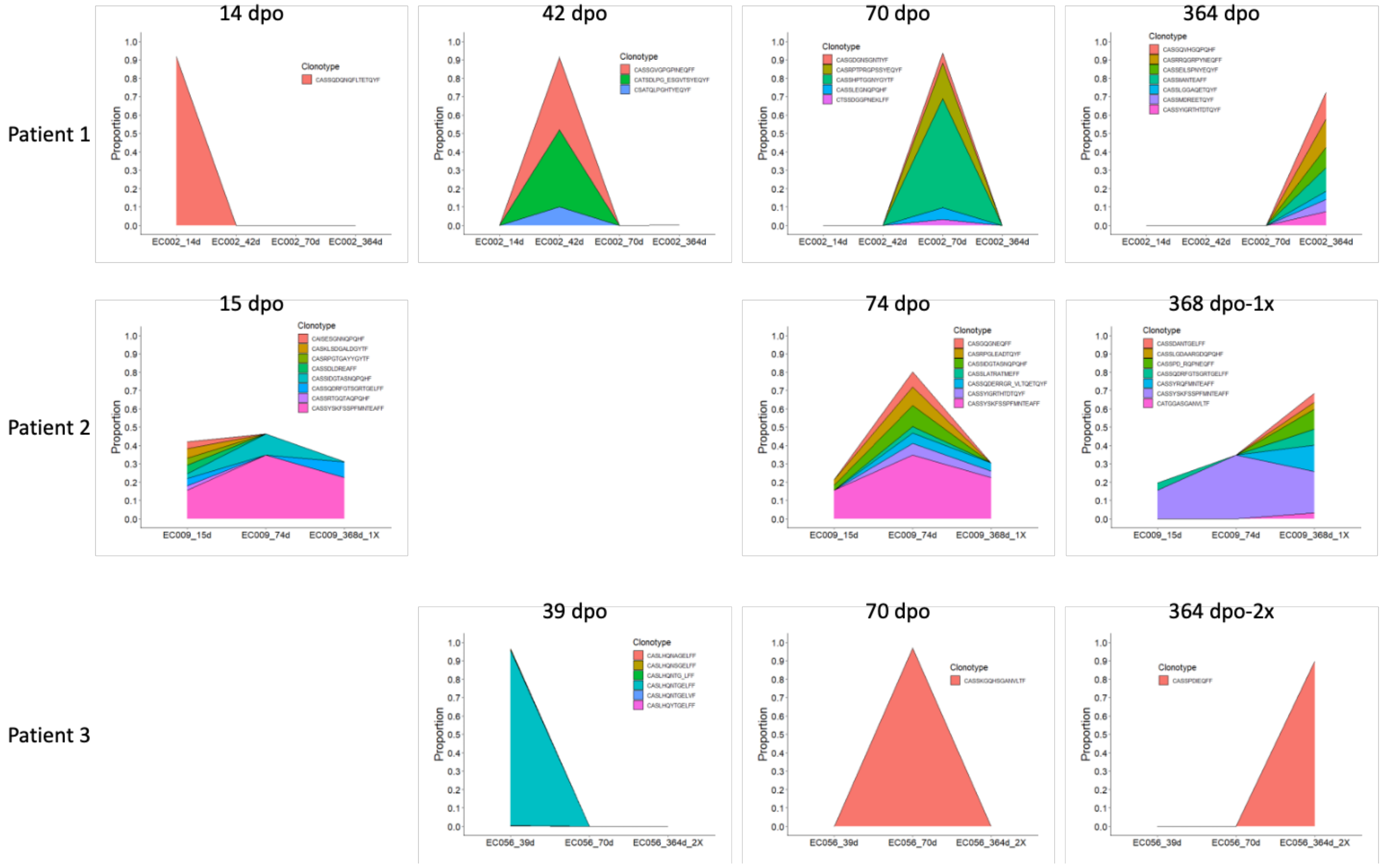

Suppl. Fig. S6. Comparison of clonotype proportions overtime in three different convalescent patients. Proportions of the most CDR3 frequent sequences are represented at different time points for each patient. Each color represents a different clonotype in each graph. Day PI = days post-infection. 1X = one vaccination dose, 2X = two vaccination doses.
